# Identification of platelet-derived biomarker in a cohort of Parkinson's disease

**DOI:** 10.1016/j.gendis.2026.102171

**Published:** 2026-04-02

**Authors:** Zhao-Ying Dong, Yu Xiang, Ling Wang, Yu Ai, Xin-Yi Du, Yuan-Yuan Sun, Zhi-Hao Lin, Tong-Fei Liu, Jian-Bo Pan, Jian-Wen Deng, Ping Li, Yang Zhao, Kai Li, Yun-Chuang Sun, Zhi-Fei Zhang, Hong-Yong Wang, Xiao-Han Li, Yong Yan, Yong-An Sun, Ou-Mei Cheng, Guo-Jun Chen

**Affiliations:** a Department of Neurology, The First Affiliated Hospital of Chongqing Medical University, Chongqing 400016, China; b Department of Neurology, Peking University First Hospital, Beijing 100000, China; c Institute of Neuroscience, Fujian Provincial Key Laboratory of Neurodegenerative Disease and Aging Research, School of Medicine, Xiamen University, Xiamen, Fujian 361005, China; d The Institute for Brain Science and Disease, Chongqing Medical University, Chongqing 400016, China; e Center for Novel Target and Therapeutic Intervention, Institute of Life Sciences, Chongqing Medical University, Chongqing 400016, China; f Department of Neurology, Heze Medical College, Heze, Shandong 274000, China; g Chongqing Medical University, Chongqing 400016, China; h Chongqing Key Laboratory of Major Neurological and Mental Disorders, Key Laboratory of Major Brain Disease and Aging Research (Ministry of Education), Chongqing Medical University, Chongqing 400016, China

**Keywords:** Biomarker, Network, Parkinson's disease, Platelet, RNA, Substantia nigra

## Abstract

Evidence has suggested that platelets (PLTs) share biochemical properties with neurons. However, whether PLTs might serve as biomarkers of Parkinson's disease (PD) remains uncertain. In this study, we first analyzed RNA data from a publicly available microarray of the substantia nigra (SN) and RNA-seq data from the PLTs of newly recruited PD patients using weighted gene coexpression network analysis (WGCNA). We found that in the SN, the majority of network modules were associated with chromatin organization, energy metabolism, and synaptic signaling, whereas those in the PLTs were related to chromatin organization and DNA metabolism. Potential PLT biomarkers were then screened by comparing the differentially expressed genes (DEGs) in the significant module pathways with those identified in the SN data by microarray. Additionally, significant genes were identified using genome-wide association study (GWAS) and transcriptome-wide association study (TWAS). The resulting 20 candidate genes were further tested for consistency in single-cell RNA-seq data, followed by assessment using receiver operating characteristic curve (ROC) analysis and qPCR validation. The combination of the CRLF3 and LRRC37A2 genes exhibited the best diagnostic performance. Collectively, both PLTs and the SN were common in the chromatin organization pathway, and the diagnostic value of PLT-derived CRLF3 and LRRC37A2 genes deserves further large-scale studies.

## Introduction

Parkinson's disease (PD), a prevalent neurodegenerative disorder,^1^ is characterized by the intracellular accumulation of α-synuclein and the loss of dopaminergic neurons in the substantia nigra (SN).^2^ Currently, the diagnosis of PD remains challenging because clinical symptoms are not typical, and diagnostic tests or biomarkers do not enable PD identification at the earliest stages.^3^ Integrated transcriptome data in the SN provide information on genes directly associated with PD progression.^4^ However, the inability to collect brain tissue from living patients prevents the use of the SN as a diagnostic biomarker. Along with technological advances, magnetic resonance imaging (MRI) and α-synuclein radiotracer imaging have been developed with various degrees of diagnostic potential.^5^ In addition to cerebrospinal fluid and the gut microbiota, blood samples could serve as a source of biomarkers for PD patients.^6^

Platelets (PLTs) are derived from budding megakaryocytes without a nuclear structure. Megakaryocytes transfer genetic information and functional nuclear constituents to circulating PLTs.^7^^,^^8^ These anucleate cells share biochemical properties with neurons,^9^ and their interactions with neutrophils contribute to inflammation and neuronal damage.^10^ In PD, PLTs decrease in number but increase in volume, with a compromised uptake of dopamine.^11^, ^12^, ^13^ Hypercoagulation of PLTs is correlated with high levels of circulating proinflammatory cytokines.^14^ Accumulating evidence highlights the potential diagnostic value of PLTs for tumors,^15^^,^^16^ cardiovascular and immune diseases,^17^ and Alzheimer's disease.^18^ However, whether molecular alterations in PLTs might serve as potential biomarkers for PD has not been well established.

Weighted gene coexpression network analysis (WGCNA) is a systems biology method for analyzing the correlation patterns of genes within and across different tissues.^19^ In this study, publicly available microarray data from the SN of PD patients and transcriptome data from the PLTs of PD patients recruited at Chongqing Medical University were collected. Significant modules in the SN and PLT were independently and jointly screened for gene significance and connectivity. The potential PLT biomarkers were then analyzed by comparing the DEGs in the PLTs with those in the SN data by microarray and in the brains of PD patients who were identified by large-scale genome-wide association study (GWAS) and transcriptome-wide association study (TWAS), resulting in the identification of 20 candidate genes. These genes were initially tested for consistency in newly available single-cell RNA-seq data. ROC analysis and final qPCR then performed using a validation set revealed that a combination of CRLF3 and LRRC37A2 genes exhibited excellent diagnostic performance.

## Materials and methods

### mRNA analysis of the substantia nigra (SN) from a publicly available database

To obtain mRNA data from the SN of PD patients and healthy controls (HCs), we searched the National Center of Biotechnology Information (NCBI) and European Bioinformatics Institute (EMBL-EBI) databases.^20^ The search keywords used were “Parkinson”, “transcriptome”, and “patients” for NCBI and “*Homo sapiens*” and “RNA array or RNA sequencing” in combination with “Parkinson” filtered by SN for EMBL-EBI. In total, 15 SN datasets were found before January 22, 2023. Of these, only one contained RNA-seq data (GSE136666),^21^ with a small sample size (5 PD patients and 5 HCs). The remaining 14 microarray datasets were selected for further analysis.

**Table 1:** Summary of substantia nigral mRNA expression profile from Internet databases in Parkinson's disease.

GEO Accession	GPL	Tissue	Samples (PD/HC)	Method	Platform Description	Cause of removal
GSE7621	GPL570	SN	25 (16/9)	Microarray	[HG-U133_Plus_2] affymetrix human genome U133 plus 2.0 array	
GSE8397(Lateral)	GPL96	Lateral SN	16 (9/7 (5))	Microarray	[HG-U133A] affymetrix human genome U133A array	(2 samples had abnormal data)
GSE26927	GPL6255	SN	20 (20/0)	Microarray	Illumina humanRef-8 v2.0 expression beadchip	No control group
GSE20333	GPL201	SN	12 (6/6)	Microarray	Affymetrix human HG-focus target array	
GSE20164	GPL96	SN	11 (6/5)	Microarray	Affymetrix human genome U133A array	
GSE20292	GPL96	SN	29 (11/18)	Microarray	Affymetrix human genome U133A array	No DEGs
GSE20163	GPL96	SN	17 (8/9)	Microarray	Affymetrix human genome U133A array	
GSE54282	GPL17047	SN	6 (3/3)	Microarray	Affymetrix human gene 1.0 ST array	Incomplete data
GSE49036	GPL570	SN	28 (20/8)	Microarray	Affymetrix human genome U133 plus 2.0 array	
GSE42966	GPL4133	SN	15 (9/6)	Microarray	Agilent-014,850 whole human genome microarray 4 × 44 K G4112F (feature number version)	Not affymetrix
GSE20295	GPL96	SN	29 (14 (11)/15)	Microarray	Affymetrix human genome U133A array	(3 samples had abnormal data)
GSE20159	GPL6947	SN	33 (16/17)	Microarray	Illumina HumanHT-12 V3.0 expression beadchip	Not affymetrix
GSE7307	GPL570	SN	13 (4/9)	Microarray	Affymetrix human genome U133 plus 2.0 array	Abnormal data
GSE136666	GPL11154	SN	10 (5/5)	RNA-seq	Illumina HiSeq 2000 (Homo sapiens)	RNA-seq
GSE114517	GPL18573	SN	28 (16/12)	RNA-seq	Illumina NextSeq 500 (Homo sapiens)	Ribosomal-RNA

The following criteria were applied for sample collection. 1) Microarray data were quantified using the Affymetrix platform. 2) The sample sizes of the cases and controls were ≥ 3.3) Differentially expressed genes were presented. 4) Data samples were collected without any chemical or physical interventions. 5) Data samples were obtained from human brain tissue at autopsy. 6) Datasets with errors were excluded (e.g., microarray data cannot be downloaded owing to incomplete uploading). Finally, 7 datasets, GSE7621,^22^ GSE8397,^23^ GSE20333,^24^ GSE20164,^24^ GSE20163,^24^ GSE49036,^25^ and GSE20295,^26^ comprising 76 PD patient samples and 57 HC samples, were obtained (Table 1). Basic information regarding cognitive function and ApoE alleles was missing in all 7 studies, whereas age and sex information was provided in four studies. The available data showed that the average ages of the PD patients and HCs were 78.36 ± 5.89 (36 samples) and 72.76 ± 10.61 years (39 samples), respectively. Some studies provided only age. A total of 16 females were included in the PD group (45 samples), and 17 females were included in the HC group (44 samples).

The microarray data were first normalized using the “Affy” package (v1.74.0) to eliminate expression variations caused by experimental techniques and to more accurately determine differences in biological functions. Logarithmic processing was then applied to facilitate symmetrical data distribution and avoid the influence of zero values. The robust multiarray average (RMA) method was used for background correction (removing the background noise during chip signal collection), quantile normalization (eliminating the nonbiological differences among samples), and summary (obtaining gene expression levels).^27^^,^^28^ The Hugo Gene Nomenclature Committee (HGNC) was used to check and update the gene symbols with HGNChelper (v0.3.1).^29^ Finally, RankProd (v3.22.0) was used to convert data values to relative gene expression levels, thus allowing for the measurement of DEGs under different experimental conditions.^30^^,^^31^

### Patients and healthy donors in the present study

It has been suggested that at least 12 high-quality replicates per condition allow for the identification of ≥ 90% of the truly differentially expressed genes (DEGs).^32^ Patients with PD and atypical Parkinson's disease (APD) and age- and sex-matched healthy controls (HCs) were recruited from the Department of Neurology outpatient clinic in the First Affiliated Hospital of Chongqing Medical University from March 2022 to June 2023. The diagnosis of PD/APD was made by movement disorder specialists according to the clinical diagnostic criteria of the Movement Disorder Society-Sponsored Revision of the Unified Parkinson's Disease Rating Scale (MDS-UPDRS).^33^ The patient exclusion criteria were as follows: 1) presence of additional neurological or psychiatric disorders other than the aforementioned diseases; 2) intake of antipsychotic drugs or anticoagulants within 1 month; 3) any acute illness with an abnormal blood count; 4) various infections, such as those of the upper respiratory tract, that occurred within 1 month; 5) a history of hematological diseases and tumors; or 6) a family history of neurological or psychiatric disorders. To exclude the possibility that an HC might develop early symptoms of PD, an additional PD screening questionnaire^34^ was administered (Table 2). To keep the number of false-positive PD cases as low as possible, the enrolled PD patients were followed up before the beginning of the analysis. In total, 22 PD patients, 13 APD patients, and 27 HC individuals were ultimately included for mRNA-seq analysis. For APD, only progressive supranuclear palsy (PSP) and multiple system atrophy (MSA) were identified and included. In the validation set,^35^ an additional 10 HC, 10 PD, and 10 APD samples were collected for qPCR analysis. The study was approved by the Research Review Board (2022–56 and 2023–416), and all the subjects provided written informed consent before participation.

**Table 2:** Basic characteristics of enrolled samples.

Basic characteristics	Health control (HC) (*n* = 27)	Parkinson's disease (PD) (*n* = 22)	Atypical Parkinson's disease (APD) (*n* = 13)	*p* value
Age, years (SD)	62.44 (8.772)	63.59 (7.920)	64.15 (9.227)	0.813
Sex, male (number, %)	12 (44.4%)	9 (40.9%)	5 (38.5%)	0.932
Disease duration, years (25%, 75%)		1.50 (1.00, 3.00)	1.50 (0.75, 3.35)	0.880
Hoehn and Yahr		2.00 (1.88, 2.50)	2.00 (2.00, 2.75)	0.113
Clinical findings (number, %)				
Resting_tremor		19 (86.4%)	4 (30.8%)	0.002
Bradykinesia		15 (68.2%)	13 (100.0%)	0.062
Rigidity		21 (95.5%)	10 (76.9%)	0.134
Asymmetric		19 (86.4%)	4 (30.8%)	0.383
Hyposmia		21 (95.5%)	5 (38.5%)	0.000

There were no statistically significant differences in the gender and age of the samples among three groups. All the enrolled PD patients were mainly in the early stage. Asymmetric rigidity and resting tremor were the main motor symptoms.

### Isolation and purification of PLT

Blood samples (10 mL) from each participant were collected into a BD vacuum vessel containing EDTA anticoagulant. The samples were stored at 4 °C immediately and processed within 6 h. Whole blood samples were centrifuged at 120 g for 20 min, resulting in the formation of PLT-rich plasma (PRP) in the supernatant. Nine-tenths of the supernatant was aspirated to avoid the potential contamination of other blood cells underneath. The resulting samples were further centrifuged at 360 g for 20 min, after which the PLTs in the pellets were dissolved in 500 μL TRIzol (Thermo Fisher Scientific) and stored at −80 °C for further use.^16^

### RNA extraction of PLT, sequencing and data analysis

Total RNA was quantified using a NanoDrop and Agilent 2100 Bioanalyzer (Thermo Fisher Scientific, MA, USA). Samples that met the following criteria were used for the subsequent assays: 1) total amount of RNA >200 ng and 2) an RNA integrity number (RIN) ≥ 7.^36^ mRNA was purified using oligo (dT)-attached magnetic beads and then fragmented into small pieces to generate a cDNA library through polymerase chain reaction (PCR). The final library was subsequently sequenced on a BGIseq500 platform (BGI-Shenzhen, China).^37^ The sample inclusion and exclusion criteria are shown in Figure 1. A total of ∼15,000 genes were detected in the PLT of patients and controls, with no overall difference among the groups. Correlation analysis of RNAs between PLTs in our study and other blood cell types, including immune cells, from public data revealed that PLT samples were not significantly contaminated (Fig. 2).^16^^,^^38^

**Figure 1:**
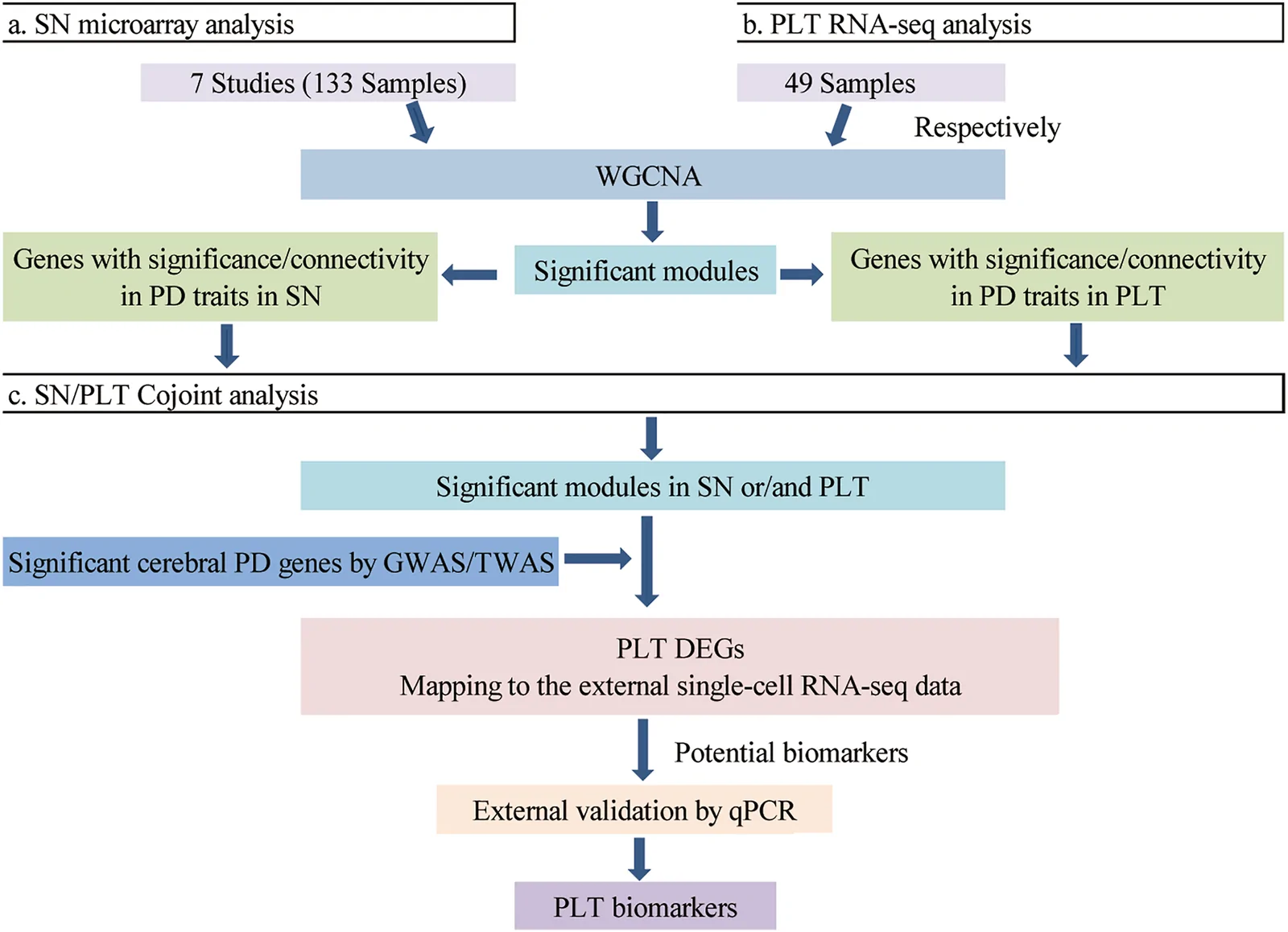
Overview of the study design. Significant modules in the substantia nigra (SN) and platelets (PLTs) associated with Parkinson's disease (PD) traits were assessed by weighted gene coexpression network analysis (WGCNA). Differentially expressed genes (DEGs) in these modules of PLTs are mapped to those in the SN and important genes in PD by large-scale genome-wide association study (GWAS) and transcriptome-wide association study (TWAS), leading to the identification of candidate PLT biomarkers. These candidate DEGs were primarily evaluated using single-cell RNA-seq data, assessed by receiver operating characteristic (ROC) curve analysis, and finally validated by quantitative real-time polymerase chain reaction (qPCR) in an external dataset.

**Figure 2:**
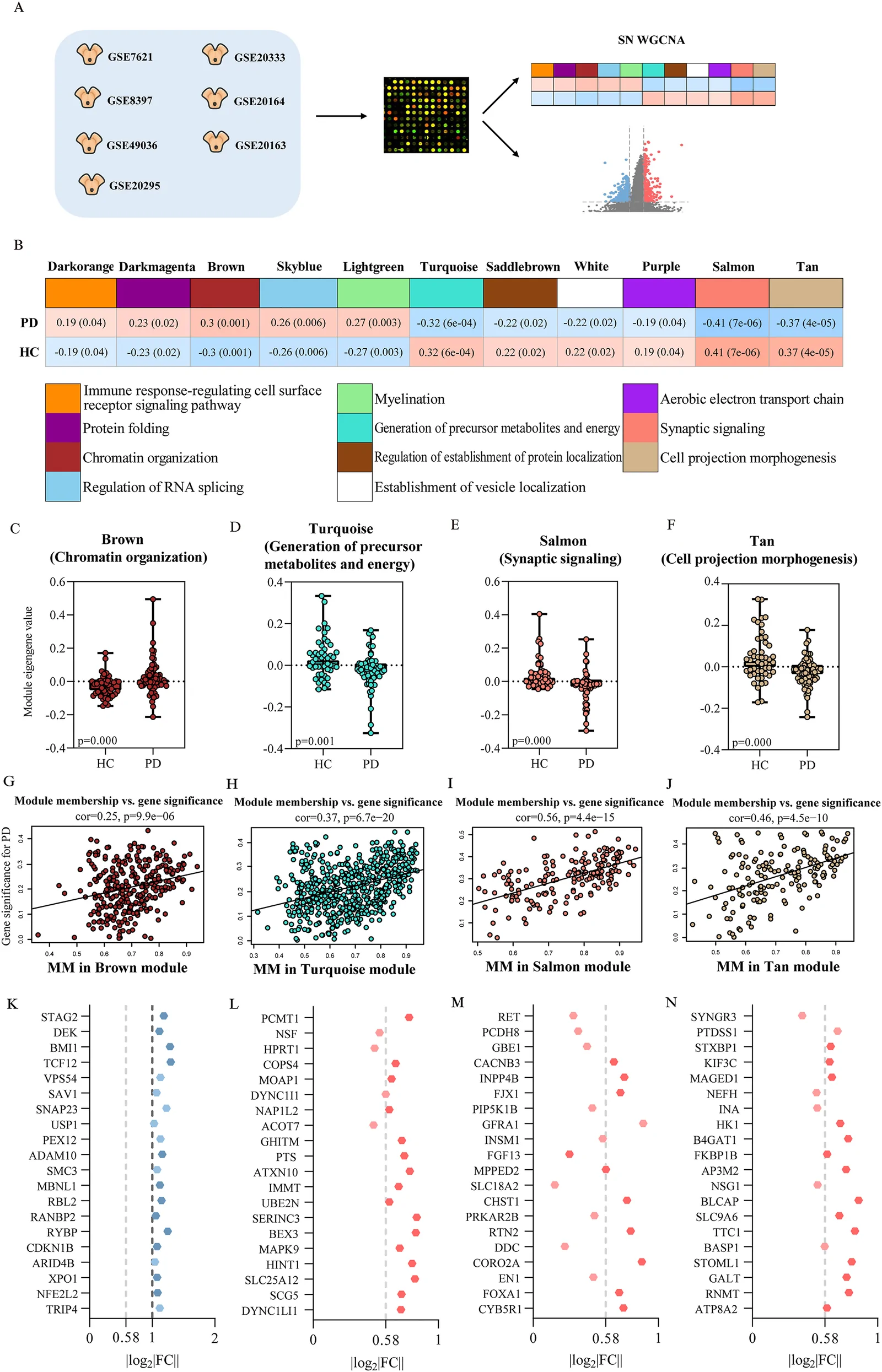
Analysis of the mRNA network in the substantia nigra (SN). **(A)** Workflow of SN microarray data collection from 7 different sources with the indicated GSE numbers. **(B)** Correlation network consisting of 11 mRNA modules significantly associated with Parkinson's disease (PD) traits. Gene Ontology Biological Process (GO-BP) analysis identifies biological processes corresponding to each module. **(C–F)** Comparison of differences in module eigengene (ME) values between the HC and PD groups. **(G**–**J)** Scatter plots showing that the absolute values of gene significance (GS) for each PD network module are significantly correlated with module membership (MM). Cor, Pearson correlation coefficient. **(K–N)** Differences in expression levels (|Log_2_ FC|) of the top 20 hub genes in each module. Red nodes: Compared with that in the HC group, the gene expression level in the PD group was upregulated. Dark blue, significantly down-regulated; dark red, significantly up-regulated. Light blue/red, no significant difference in the gene expression level.

SOAPnuke (v1.5.2) was first used for raw data processing.^39^ 1) Reads containing sequencing adapters were removed. 2) Reads with a low-quality base ratio (base quality less than or equal to exceeding 20% were removed. 3) Reads with an unknown base (‘N' base) ratio greater than 5% were removed. All sequencing data were analyzed on the Illumina platform and filtered using Fastp (v0.23.0) to achieve greater comparability among the samples.^37^ Clean reads were obtained and stored in FASTQ format^40^ and aligned to the NCBI GCF_000001405.39_GRCh38. p13 version of the *H. sapiens* reference genome using Bowtie 2 (v2.2.5).^41^ The RSEM (v1.2.12) package was used to calculate gene expression levels, which were presented as fragments per kilobase million (FPKM).^42^ DESeq2 (v1.4.5) was used for DEG analysis,^43^ and a fold change (FC) > 0.58 and *p* < 0.05 were applied.

### Bioinformatics

The WGCNA package (v1.72) was used to identify modules and genes strongly correlated with clinical features.^19^ For data input and cleaning, the “limma” package (v3.52.4) was used to correct for batch effects on the 133 SN microarray samples, which were clustered after removing obvious outliers, resulting in 115 samples that were included in the final analysis. For PLT samples from PD patients and HCs, the median absolute deviation (MAD) was used to filter low-expressing or nonvarying genes, which were considered proxies for noise, and the top 10,000 MAD genes were selected. To further test the consistency of the DEGs screened for potential biomarkers in PLT, we additionally analyzed the neuronal RNA-seq data (GSE243639), which included those from 15 PD (Braak stage 2.9) and 14 HC patients, with average ages of 82.33 and 75.50 years, respectively.^44^ A soft threshold (*β* value) was applied to the data analysis, with *β* = 12 in the microarray and neuronal RNA-seq data and *β* = 6 in the PLT RNA-seq data, which could be relevant as previously reported.^45^^,^^46^

Module eigengene (ME, the first principal component of the gene expression matrix in a module), gene significance (GS, association strength between a gene and PD trait calculated by the absolute value), and module membership (MM, the correlation between genes and ME measured by correlation efficiency) were used to identify the potentially important genes. Gene Ontology Biological Process (GO-BP) pathway analysis was performed to assess the potential biological functions of the module genes.^47^ The maximum clique centrality (MCC) in Cytohabba/Cytoscape (v3.9.1) was used to calculate and plot the top 20 genes in each module that were most highly connected to other genes. The ggcorrplot (v1.0.12) and pheatmap (v0.1.4.1) packages were used for Pearson correlation coefficient calculations and correlation matrix generation. Metascape (http://Metascape.org) and DAVID (v6.8) (https://david.ncifcrf.gov/) were used to search for pathways and enriched genes in the significant modules.

### Quantitative real-time polymerase chain reaction

Total RNA was isolated using the platelet precipitation method. The target genes were amplified using qPCR primers (Table S8) and reverse transcription kits (SynScript®III RT SuperMix, TSK314S; Tsingke, China). cDNA obtained through reverse transcription was diluted 2x and used as the template for qPCR amplification (ArtiCanCEO SYBR qPCR Mix, TSE401, China).

### Statistical analysis

Statistical analysis was performed using IBM SPSS Statistics (v26.0; IBM Corp., Armonk, NY, USA). Categorical variables are expressed as percentages and were analyzed using the chi-square test. The Kolmogorov–Smirnov test was used to determine the normality of continuous variables. Age was normally distributed and is presented as the mean ± standard deviation (mean ± SD) and was analyzed using one-way analysis of variance (ANOVA). The Mann–Whitney *U* test was applied to compare disease duration and Hoehn and Yahr (H&Y) stage between the two groups. A receiver operating characteristic (ROC) curve was used to measure the sensitivity and specificity of the selected genes. The Youden index was used to calculate the cutoff value. A *p* < 0.05 was considered statistically significant.

## Results

### Study design

WGCNA is an R package that is used to identify highly correlated genes that form clusters (modules).^19^ This method allows the identification of hub genes within and across different tissue samples.^43^^,^^48^ Analysis of gene expression profiles revealed significant modules in the SN and PLT, respectively. Both the common and PLT-specific DEGs in the significant module pathways were extracted as potential PLT biomarkers. To overcome the potentially limited reliability of the microarray data, we additionally identified significant genes from large-scale GWAS/TWAS data that were significantly altered in PD brains and thus were mapped to DEGs in PLT. The diagnostic value of the resulting DEGs was then assessed using the area under the curve (AUC), leading to the identification of potential PLT biomarkers, which were ultimately validated by qPCR in an independent sample set (Fig. 1). Upon completion of our data analysis, single-cell RNA-seq data from the SN of PD patients were available, allowing PLT-derived DEGs to be further tested in SN neurons (Fig. 1). Thus, the assessment of PLT biomarkers in this cohort of PD patients in the present study involved a comparison of DEGs in the microarray, GWAS/TWAS, and single-cell RNA-seq data followed by validation via qPCR.

### Network module enrichment in the SN of PD

We analyzed 76 PD samples and 57 HC samples from 7 public datasets through WGCNA (Fig. 2A). In total, 1269 down-regulated genes and 1428 up-regulated genes were obtained. As shown in Figure 2B, the RNA network consisted of 38 gene co-expression modules, of which 11 were significantly associated with PD phenotypes. Subsequent GO-BP analysis of each module revealed that the PD traits were positively related to the immune response-regulating cell surface receptor signaling pathway, protein folding, chromatin organization, and the regulation of RNA splicing and myelination. Pathways that were negatively correlated with PD included the generation of precursor metabolites and energy, regulation of protein localization, establishment of vesicle localization, aerobic electron transport chain, synaptic signaling, and cell projection morphogenesis (Fig. 2B and Table S1).

We then assessed the module eigengene (ME, the first principal component) in these significant modules (Table S1). In the top 4 modules (Fig. 2C–F), the ME value for chromatin organization (brown) significantly increased, whereas that for the generation of precursor metabolites and energy (turquoise), synaptic signaling (salmon), and cell projection morphogenesis (tan) significantly decreased in PD patients. Accordingly, the absolute value of gene significance (GS), which indicates the strength of the association between a gene and PD trait, was significantly correlated with MM (module membership), which was calculated by correlation efficiency (cor) (Fig. 2G–J), indicating that these module genes were significantly associated with the PD phenotype. The top 20 genes in each module were further selected for DEG assessment. As shown in Figure 2K–N, most genes were significantly down-regulated (dark blue) or up-regulated (dark red) in PD, as indicated by the absolute value of fold change (log_2_FC). To further understand the connectivity between genes in the selected top 4 modules, a gene network was constructed, which led to a separate set of DEGs. The top 20 genes with high connectivity and DEG values are presented in each module (Fig. S3).

### Network module enrichment in PLT

In total, 1126 genes were down-regulated and 223 genes were up-regulated in the PLT of PD patients compared with those of HCs (Fig. 3A and Table S2). WGNCA revealed a total of 8 significant modules (Fig. 3B and Table S3), which were associated with the PD phenotype, disease duration, and H–Y stage but not with sex or age (Fig. 3B and Table S3). The top 4 modules included the chromatin organization (red and black), innate immune response (pink), and DNA metabolic process (green) modules, in which the MEs were significantly decreased in PD patients compared with those in HCs (Fig. 3C–F). Notably, the chromatin organization and immune response modules were also found in the SN, indicating that these RNA networks were partially preserved. The absolute value of gene significance (GS) relative to the PD phenotype (Fig. 3G–J) and H–Y stage (Fig. 3K–N) was correlated with MM, supporting a close association of these genes with PD. As shown in Figure 3O–R, the top 20 DEGs in PD patients compared with HCs were included, with relative alterations in the absolute value of log2FC, in which the down-regulation and up-regulation are shown as red/blue, respectively. Similarly, the top 20 genes in the 4 modules with high connectivity were selected for network construction and DEG analysis (Fig. S4), which might provide insight for further understanding the hub genes.

**Figure 3:**
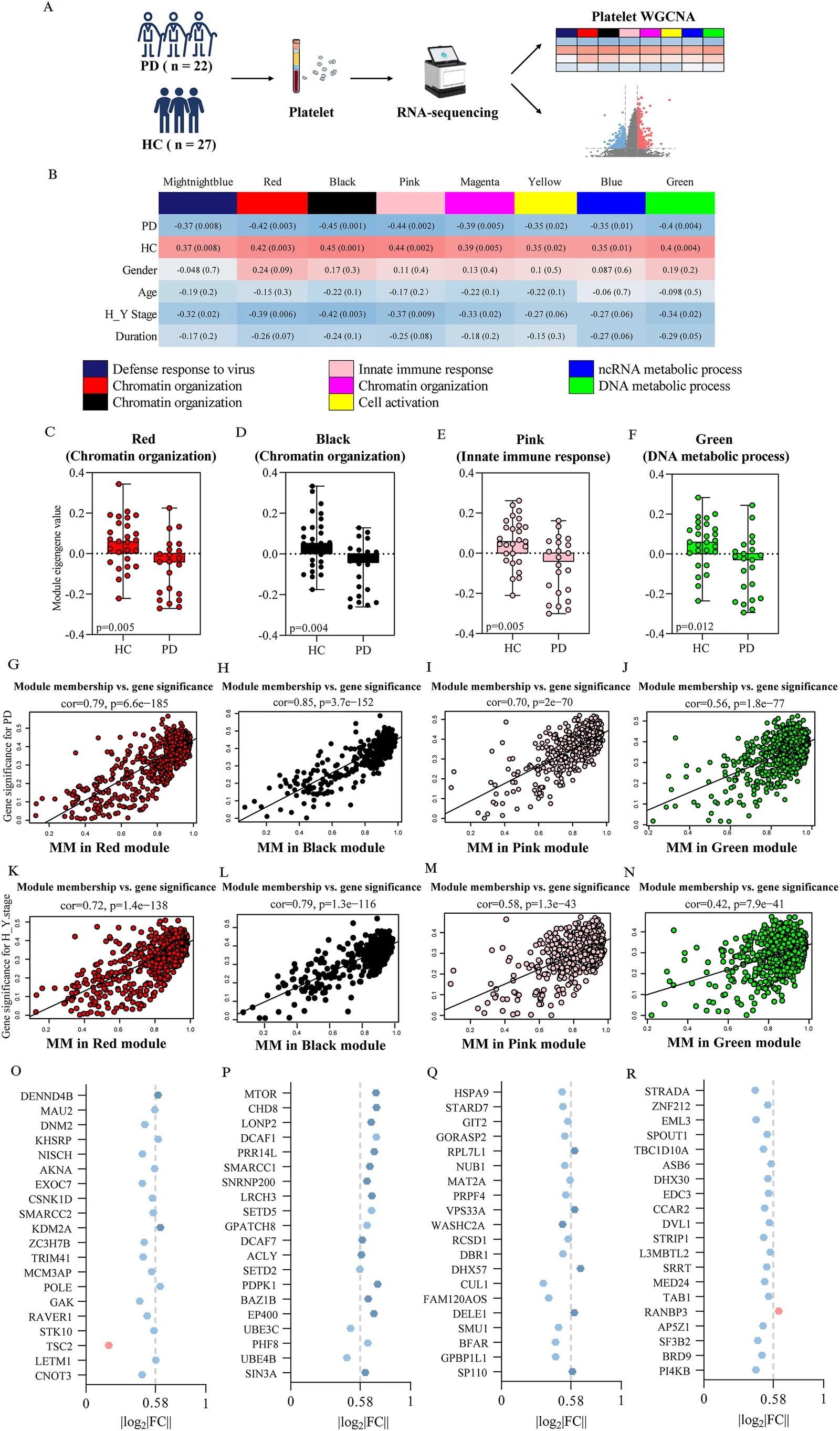
RNA network analysis of platelets (PLTs). **(A)** Workflow of PLT transcriptome analysis from Parkinson's disease (PD) patients and healthy controls (HCs) enrolled in this study. **(B)** A correlation network consisting of 8 mRNA modules significantly associated with PD traits but not sex, age, or disease duration. Six modules were significantly associated with the Hoehn–Yahr stage. Gene Ontology Biological Process (GO-BP) analysis revealed biological processes corresponding to each module. **(C–F)** Comparison of differences in ME values between HCs and PD patients. **(G**–**J)** Scatter plots showing that the absolute values of gene significance (GS) for PD in the network module are significantly correlated with module membership (MM). **(K–N)** Scatter plots showing that the absolute values of gene significance (GS) for the Hoehn–Yahr stage in the network module are significantly correlated with module membership (MM). **(O**–**R)** Differences in expression levels (|Log_2_ FC|) of the top 20 hub genes in each module. Dark blue, significantly down-regulated; light blue, no significant difference in gene expression level.

### Module pathways that were common in the SN and PLT

PLT activity is closely associated with neurodegenerative diseases.^49^ To understand the functional connection between PLT and the SN, we analyzed the genes enriched in 11 SN and 8 PLT modules that were significantly associated with PD. As shown in Figure 4A, some module-enriched genes overlapped between the SN and PLT. Furthermore, module pathway analysis using GO-BP revealed that the most significant pathways included chromatin organization and the DNA damage response, whereas organelle localization, membrane organization, and intracellular protein transport were also common in the SN and PLT (Fig. 4B). These results indicated that some key pathways in the SN were also present in PLT, thus allowing us to analyze PLT-derived biomarkers.

**Figure 4:**
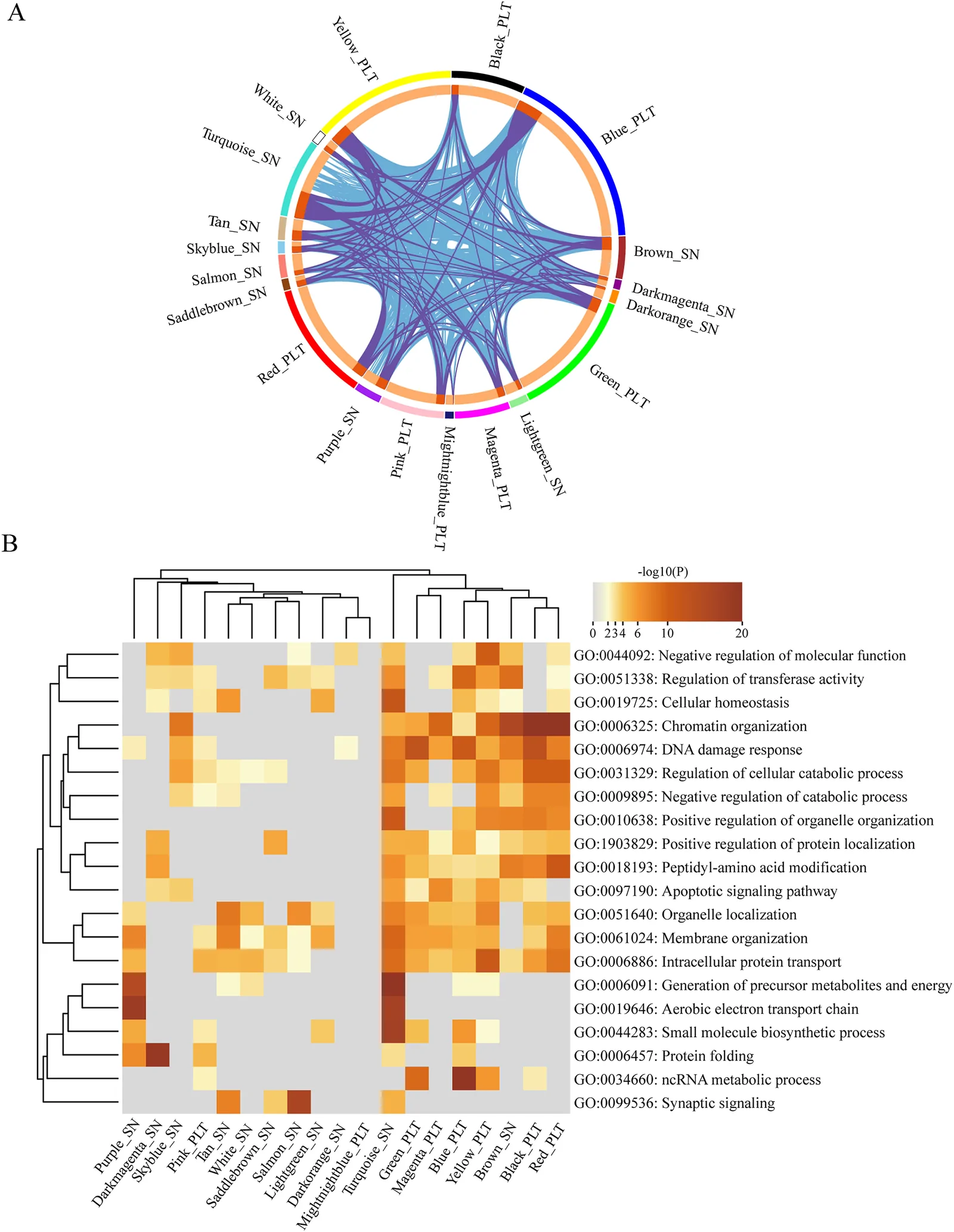
Module genes that partially overlap between the platelets (PLTs) and substantia nigra (SN). **(A)** In modules significantly associated with Parkinson's disease (PD) traits, genes and Gene Ontology Biological Process (GO-BP) pathways in PLTs and the SN (STN) partly overlap. On the outside, arcs with the indicated colors represent different SN and PLT modules corresponding to those shown in Figure 2, Figure 3, respectively. The inside circle shows dark and light orange arcs; each arc represents a collection of module genes corresponding to the outside. Dark oranges connect genes that overlap with those in other modules (purple lines), whereas blue lines link genes associated with the GO-BP pathways shared by these overlapping genes. Light orange represents genes that lack functional connections. **(B)** Major pathways enriched in PLT or SN modules that are significantly associated with PD. Terms representing the different modules in SN and PLT are shown in the bottom panel, whereas the top 20 GO-BP pathways are indicated on the right side. The darker the color, the more significant the pathways in the module.

### Identification of the candidate biomarkers in PLT

To explore potential PLT-driven biomarkers, we first analyzed the DEGs in the 8 clustered modules of PLT that were mapped to those in the SN modules. As shown in Figure 5A, a total of 26 DEGs were coexpressed in the significant SN and PLT modules. The resulting 12 genes showed a consistent trend (Table S4) and could be categorized into the following two modules (Fig. 5B): cell activation (EPB41L2, BACH2, ZNF217, and PNN) and chromatin organization (ATXN1, PCF11, CRLF3, RBL2, MED23, MAP4, ARHGEF2, and SMARCC1). Accordingly, the relative expression levels of these genes were significantly lower in the SN and PLT of PD patients than in HCs (Fig. 5C).

**Figure 5:**
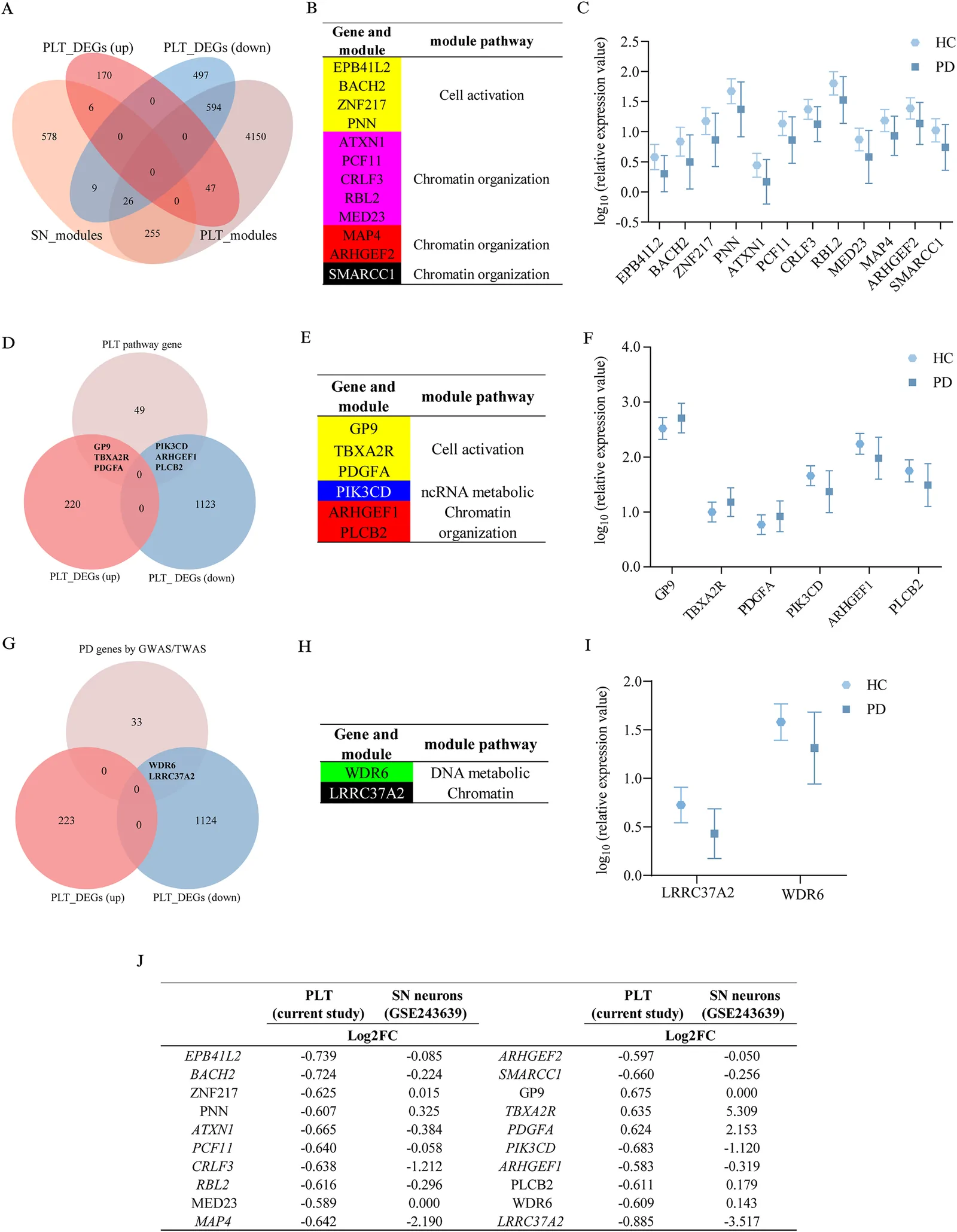
Candidate platelets (PLTs) biomarkers and primary testing. **(A**–**C)** Differentially expressed genes (DEGs) in network modules of PLTs are first mapped to those in the substantia nigra (SN). Venn diagram showing 26 module genes that overlap (A), in which 12 genes with the corresponding modules are consistently changed between the SN and PLTs (B) and are significantly altered in the PLTs of Parkinson's disease (PD) patients (C). (F–F) DEGs in network modules of PLTs are mapped to “platelet” pathway genes that are not enriched in the SN (referring to PLT-specific genes). Venn diagram showing six overlapping genes with the corresponding modules **(D, E)**, whose expression is significantly altered in the PLTs of PD patients **(F)**. **(G**–**I)** DEGs in network modules of PLTs are mapped to 33 characteristic PD genes by large-scale genome-wide association study (GWAS) and transcriptome-wide association study (TWAS). Venn diagram showing two overlapping genes (WDR6 and LRRC37A2) with the corresponding modules (G, H), whose expression is significantly altered in the PLTs of PD patients (I). **(J)** The resulting 20 genes in PLTs were primarily tested in neurons of the SN by single-cell RNA-seq (GSE243639). Most of the genes were consistently altered.

Because of the unique biological characteristics of PLT, some PLT-specific genes that were absent in the SN might also have diagnostic value. We next used the keyword “platelet” as an additional strategy for pathway building, which led to the identification of 49 genes mainly enriched in PLT activation, aggregation, and PLTα particle-related pathways (Table S4). Through DEG mapping (Fig. 5D), we identified 6 genes enriched in module pathways associated with PD, including the down-regulated genes GP9, TBXA2R, and PDGFA and the up-regulated genes PIK3CD, ARHGEF1, and PLCB2 within the corresponding module pathways (Fig. 5E and F).

In addition to microarray data in the SN, large-scale, population-based GWAS and TWAS data from the past five years provide another source of genes that are significantly altered in the brains of PD patients.^50^, ^51^, ^52^, ^53^, ^54^, ^55^, ^56^ Thus, we retrieved a total of 33 characteristic PD genes (Tables S1, S3, and S6), which were mapped to DEGs in PLT (Fig. 5G). The expression of WDR6 and LRRC37A2 was significantly down-regulated in the PLTs of PD patients, corresponding to DNA metabolic processes and chromatin organization, respectively (Fig. 5H and I). Therefore, the integrated analysis led to a total of 20 candidate DEGs that showed diagnostic potential.

Upon completion of our data analysis, single-cell RNA-seq data (GSE243639) from the SN of PD patients were publicly available4^44^ (Table S8). Thus, the 20 candidate DEGs could be initially tested in this dataset. WGCNA of the SN neurons revealed 3 coexpression modules that were significantly associated with PD traits (Fig. S5A). GO-BP pathways included well-documented dopamine biosynthesis and synaptic signaling. Notably, pathways such as those related to the regulation of chromosome separation and mitotic sister chromatid segregation were also identified (Fig. S5B–S5D), suggesting that altered chromosomal processing was preserved in both the PLT and SN. All 20 DEGs were ultimately detected in the single-cell RNA data, and approximately 70% of them were consistently found in the PLT (Fig. 5J).

### The combination of the CRLF3 and LRRC37A2 genes resulted in the best diagnostic performance

The diagnostic value of the 20 genes was analyzed by the area under the curve (AUC). Each of these genes could independently achieve an ideal AUC value of 0.7, independent of age and sex (Fig. S6A). Among these genes, cytokine receptor-like factor 3 (CRLF3) and thromboxane A2 receptor (TBXA2R) showed the highest GS in the intersection module of PLT and the SN, whereas leucine-rich repeat containing 37 member A2 (LRRC37A2) alone exhibited the highest AUC value (0.840) in PD patients relative to that in HCs (Fig. S7). Therefore, we combined these three genes for PD diagnosis, leading to an AUC value of 0.867 between PD patients and HCs (Fig. S6B and Table S7). This combination of genes also differentiated PD patients from APD patients and APD patients from HCs, with AUC values of 0.804 and 0.909, respectively (Fig. S6C and S6D).

The expression levels of LRRC37A2, CRLF3, and TBXA2R were ultimately validated by qPCR in an external dataset that included 10 PD patients, 10 APD patients, and 10 HCs. As shown in Figure 6A–C, except for those of TBXA2R, the mRNA levels of CRLF3 and LRRC37A2 were consistently decreased in the SN of both PLTs (Table S9). Thus, LRRC37A2 and CRLF3 were selected for further ROC analysis. As shown in Figure 6D–F, the combination of LRRC37A2 and CRLF3 genes exhibited optimal diagnostic efficiency for differentiating PD patients from HCs, PD patients from APD patients, and APD patients from HCs, with corresponding AUCs of 0.920, 0.840, and 1.000, respectively.

**Figure 6:**
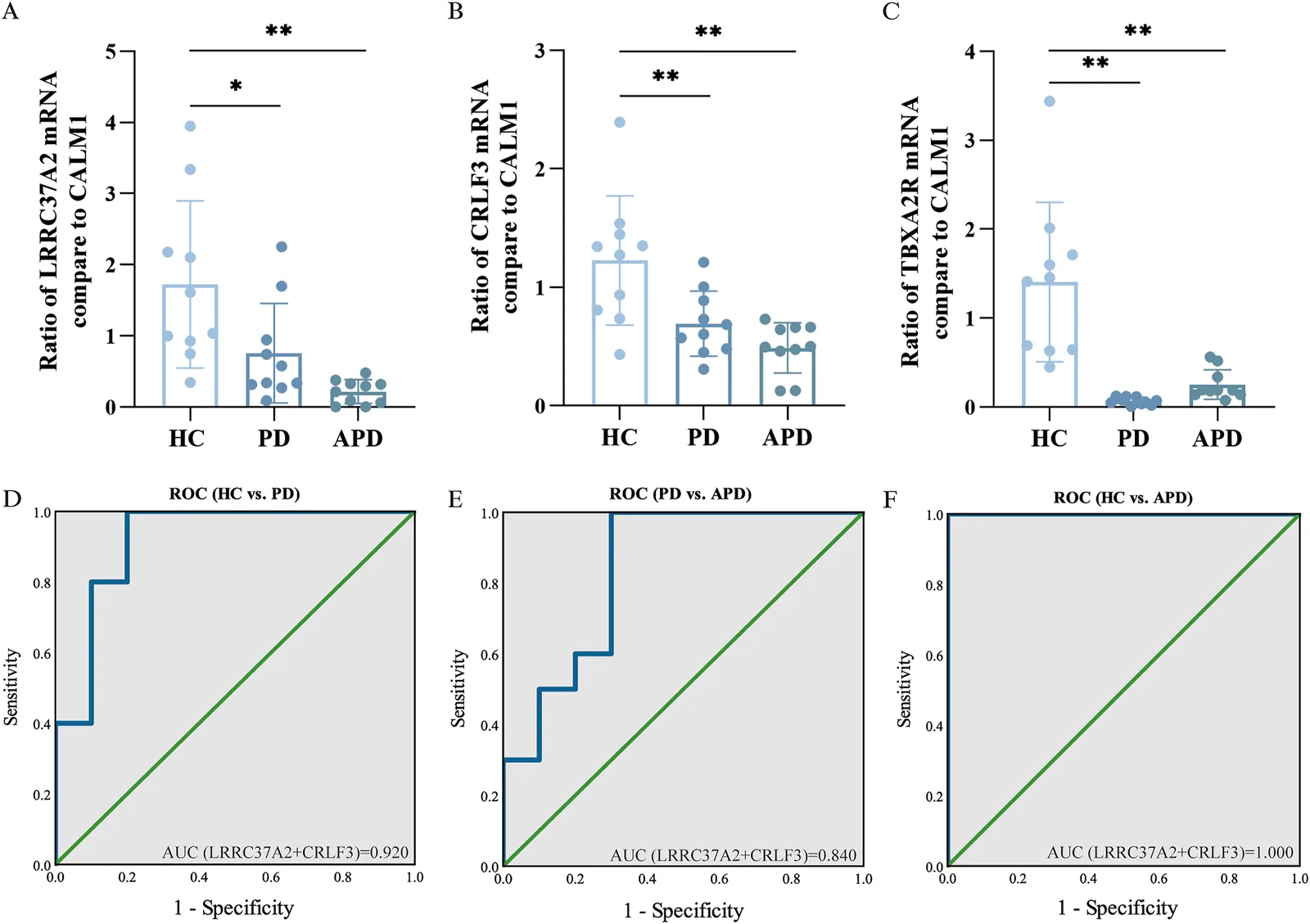
Combination of the CRLF and LRRC37A2 genes showed the best diagnostic performance. **(A**–**C)** Relative mRNA levels of LRRC37A2 (A), CRLF3 (B), and TBXA2R (C) were quantified by qPCR in an external validation set. In each of the healthy controls (HCs), Parkinson's disease (PD) and atypical Parkinson's disease (APD) groups, a total of 10 samples were collected. **(D**–**F)** Receiver operating characteristic (ROC) analysis using a combination of CRLF3 and LRRC37A2 to compare HCs *vs.* PDs (D), PDs *vs.* APDs (E) and HCs *vs.* APDs (F).

## Discussion

The major findings of this study include the following. (1) Network modules in the SN provide insights into potential molecular alterations in the pathophysiology of PD. (2) Some modules, including immune response and chromatin organization, are preserved in both the SN and PLT, suggesting that PLTs are functionally linked to the SN. (3) The combination of the CRLF3 and LRRC37A2 genes resulted in the best diagnostic performance, suggesting that PLT genes could serve as PD biomarkers.

The results of the present study revealed that the top modules in the SN of PD patients included chromatin organization, protein folding, precursor metabolites and energy, and synaptic signaling. Although the role of chromatin organization in PD is not well understood, previous studies have demonstrated that chromatin accessibility is dynamically altered during the differentiation of dopaminergic cells,^57^ whereas DNA damage mediates neurotoxicity in PD.^58^ On the other hand, it is relatively clear that neurons are sensitive to the unfolded protein response (UPR) and endoplasmic reticulum stress, which play important roles in neurodegenerative diseases.^59^^,^^60^ Metabolites and energy modules could be associated with mitochondrial dysfunction, which is involved in both monogenic and idiopathic PD.^61^^,^^62^ Disruption of mitochondrial-associated UPR and synaptic proteostasis may collectively contribute to synaptic loss.^63^^,^^64^ Our data further highlight that the network genes involved in chromatin organization are functionally linked to PD.

PLTs carry the genetic code from megakaryocytes, which includes various RNA and proteins involved in PLT functions.^8^ These cells also share transcription factors, including STAT3 and NF-kappaB.^65^^,^^66^ In particular, PD-specific proteins and miRNAs are expressed in PLT.^67^ In line with this, both common and unique modules were identified between PLT and the SN in our study. Among the common modules are immune response, chromatin organization, and DNA metabolism. Synaptic signaling and myelination, which occur in the SN, are likely absent in PLTs, whereas “platelet” pathways are specifically enriched in PLTs. These module genes in PLTs might be associated with brain activity. PLTs may regulate neuronal function through receptor interactions, exosome release, and other mechanisms that control neural precursor differentiation.^68^ For instance, youth-associated platelet factor 4 delays brain aging.^69^ The PLT receptor glycoprotein Ibalpha (GPIbα) mediates neutrophil infiltration and brain inflammation in stroke and related disorders.^70^ PLT-derived growth factor regulates neurogenesis and protects dopamine neurons in an animal model of PD.^71^^,^^72^ We speculate that module genes in PLT provide important information for identifying potential biomarkers for PD.^73^

Our study identified 20 candidate DEGs that were mapped to those in the microarray and GWAS/TWAS data, whereas large portions of these DEGs were also present in SN neurons according to single-cell RNA-seq. Although ROC analysis revealed that a combination of CRLF3, LRRC37A2, and TBXA2R has excellent diagnostic value, only CRLF3 and LRRC37A2 were validated by qPCR in the external dataset. The inconsistency of TBXA2R between RNA-seq and qPCR could be attributed to many factors.^74^ The inherent characteristics of genes (gene length, GC content, etc.), the library construction method of RNA-seq, and the quantification algorithm might lead to a biased calculation of gene expression levels.^75^ Nonetheless, these genes could be functionally linked to PD. Neuronal CRLF3 is protective and critical for neurogenesis,^76^^,^^77^ whereas PLT-derived CRLF3 controls the PLT count, which is implicated in thrombocythemia.^78^ Moreover, an increased copy number of LRRC37A2 is associated with a reduced risk of PD, and astrocytic LRRC37A2 interacts with α-synuclein and colocalizes with Lewy bodies in the SN of PD patients.^79^ Therefore, the best diagnostic performance of CRLF3 and LRRC37A2 in differentiating PD patients from HCs might deserve further large-scale investigation.

The limitations of the study include the following. First, comparing data from sequencing and microarray platforms could lead to errors and artifacts. Indeed, only some of the 20 candidate genes showed consistent changes in the single-cell RNA-seq data. Second, due to incomplete demographic information, we were unable to completely clarify the effects of other factors on gene expression profiles, such as cognition, and other aspects of the clinical trajectory of Parkinson's disease. Third, the PLT sample size is relatively small, which may result in potential bias. Nonetheless, our meta-analysis of the microarray data contributes to the identification of important modules in the SN and their relationships with molecular alterations in PLTs, where some module genes, including CRLF3 and LRRC37A2, provide valuable insights for therapeutic intervention.

## CRediT authorship contribution statement

**Zhao-Ying Dong:** Writing – review & editing, Writing – original draft, Methodology, Investigation, Formal analysis, Data curation, Conceptualization. **Yu Xiang:** Software, Methodology, Data curation. **Ling Wang:** Software, Formal analysis, Data curation. **Yu Ai:** Writing – original draft, Validation, Formal analysis. **Xin-Yi Du:** Software, Methodology, Formal analysis, Data curation. **Yuan-Yuan Sun:** Data curation. **Zhi-Hao Lin:** Formal analysis, Data curation. **Tong-Fei Liu:** Methodology, Data curation, Conceptualization. **Jian-Bo Pan:** Visualization, Supervision, Investigation. **Jian-Wen Deng:** Conceptualization. **Ping Li:** Data curation. **Yang Zhao:** Data curation. **Kai Li:** Data curation. **Yun-Chuang Sun:** Data curation. **Zhi-Fei Zhang:** Data curation. **Hong-Yong Wang:** Data curation. **Xiao-Han Li:** Data curation. **Yong Yan:** Supervision, Data curation, Conceptualization. **Yong-An Sun:** Writing – review & editing, Visualization, Funding acquisition. **Ou-Mei Cheng:** Supervision, Project administration, Investigation, Funding acquisition, Data curation, Conceptualization. **Guo-Jun Chen:** Writing – review & editing, Visualization, Supervision, Methodology, Investigation, Funding acquisition, Formal analysis, Conceptualization.

## Ethics declaration

The study was approved by the Research Review Board (2022–56 and 2023–416), and all the subjects provided informed consent forms before participation.

## Funding

This work was supported by the 10.13039/501100001809National Natural Science Foundation of China (No. 81971030, 82271461) to G-J Chen, the 10.13039/501100001809National Natural Science Foundation of China (No. 81871002) to O-M Cheng, Beijing Natural Science Foundation (No. L251024), and Key Program of National Natural Science Foundation of China (No. 12231018) to Y-A Sun.

## Conflict of interests

Dr. Guo-Jun Chen is a member of *Genes & Diseases* Editorial Board. To minimize bias, he was excluded from all editorial decision-making related to the acceptance of this article for publication. The remaining authors declare no conflict of interests.

## References

[1] Kalia L.V., Lang A.E. (2015). Parkinson's disease. Lancet.

[2] Dickson D.W. (2018). Neuropathology of Parkinson disease. Parkinsonism Relat Disord.

[3] Armstrong M.J., Okun M.S. (2020). Diagnosis and treatment of Parkinson disease: a review. JAMA.

[4] Huang J., Li B., Wei H. (2024). Integrative analysis of gene expression profiles of substantia nigra identifies potential diagnosis biomarkers in Parkinson's disease. Sci Rep.

[5] Tolosa E., Garrido A., Scholz S.W., Poewe W. (2021). Challenges in the diagnosis of Parkinson's disease. Lancet Neurol.

[6] Tönges L., Buhmann C., Klebe S. (2022). Blood-based biomarker in Parkinson's disease: potential for future applications in clinical research and practice. J Neural Transm.

[7] Bluteau O., Langlois T., Rivera-Munoz P. (2013). Developmental changes in human megakaryopoiesis. J Thromb Haemost.

[8] Rondina M.T., Weyrich A.S. (2015). Regulation of the genetic code in megakaryocytes and platelets. J Thromb Haemost.

[9] Veitinger M., Varga B., Guterres S.B., Zellner M. (2014). Platelets, a reliable source for peripheral Alzheimer's disease biomarkers?. Acta Neuropathol Commun.

[10] Chou M.L., Babamale A.O., Walker T.L., Cognasse F., Blum D., Burnouf T. (2023). Blood-brain crosstalk: the roles of neutrophils, platelets, and neutrophil extracellular traps in neuropathologies. Trends Neurosci.

[11] Rabey J.M., Shabtai H., Graff E., Oberman Z. (1993). 3H] dopamine uptake by platelet storage granules in Parkinson's disease. Life Sci.

[12] Koçer A., Yaman A., Niftaliyev E., Dürüyen H., Eryılmaz M., Koçer E. (2013). Assessment of platelet indices in patients with neurodegenerative diseases: mean platelet volume was increased in patients with Parkinson's disease. Curr Gerontol Geriatr Res.

[13] Behari M., Shrivastava M. (2013). Role of platelets in neurodegenerative diseases: a universal pathophysiology. Int J Neurosci.

[14] Adams B., Nunes J.M., Page M.J. (2019). Parkinson's disease: a systemic inflammatory disease accompanied by bacterial inflammagens. Front Aging Neurosci.

[15] In 't Veld S.G.J.G., Arkani M., Post E. (2022). Detection and localization of early- and late-stage cancers using platelet RNA. Cancer Cell.

[16] Best M.G., Sol N., Kooi I. (2015). RNA-seq of tumor-educated platelets enables blood-based pan-cancer, multiclass, and molecular pathway cancer diagnostics. Cancer Cell.

[17] Eicher J.D., Wakabayashi Y., Vitseva O. (2016). Characterization of the platelet transcriptome by RNA sequencing in patients with acute myocardial infarction. Platelets.

[18] Yu H., Liu Y., He B. (2021). Platelet biomarkers for a descending cognitive function: a proteomic approach. Aging Cell.

[19] Langfelder P., Horvath S. (2008). WGCNA: an R package for weighted correlation network analysis. BMC Bioinformatics.

[20] Sarkans U., Füllgrabe A., Ali A. (2021). From ArrayExpress to BioStudies. Nucleic Acids Res.

[21] Xicoy H., Brouwers J.F., Wieringa B., Martens G.J.M. (2020). Explorative combined lipid and transcriptomic profiling of substantia nigra and putamen in Parkinson's disease. Cells.

[22] Lesnick T.G., Papapetropoulos S., Mash D.C. (2007). A genomic pathway approach to a complex disease: axon guidance and Parkinson disease. PLoS Genet.

[23] Duke D.C., Moran L.B., Pearce R.K.B., Graeber M.B. (2007). The medial and lateral substantia nigra in Parkinson's disease: mRNA profiles associated with higher brain tissue vulnerability. Neurogenetics.

[24] Zheng B., Liao Z., Locascio J.J. (2010). PGC-1α, a potential therapeutic target for early intervention in Parkinson's disease. Sci Transl Med.

[25] Dijkstra A.A., Ingrassia A., de Menezes R.X. (2015). Evidence for immune response, axonal dysfunction and reduced endocytosis in the substantia nigra in early stage Parkinson's disease. PLoS One.

[26] Zhang Y., James M., Middleton F.A., Davis R.L. (2005). Transcriptional analysis of multiple brain regions in Parkinson's disease supports the involvement of specific protein processing, energy metabolism, and signaling pathways, and suggests novel disease mechanisms. Am J Med Genet B Neuropsychiatr Genet.

[27] Gautier L., Cope L., Bolstad B.M., Irizarry R.A. (2004). Affy: analysis of Affymetrix GeneChip data at the probe level. Bioinformatics.

[28] Irizarry R.A., Bolstad B.M., Collin F., Cope L.M., Hobbs B., Speed T.P. (2003). Summaries of Affymetrix GeneChip probe level data. Nucleic Acids Res.

[29] Oh S., Abdelnabi J., Al-Dulaimi R. (2020). HGNChelper: identification and correction of invalid gene symbols for human and mouse. F1000Res.

[30] Del Carratore F., Jankevics A., Eisinga R., Heskes T., Hong F., Breitling R. (2017). RankProd 2.0: a refactored bioconductor package for detecting differentially expressed features in molecular profiling datasets. Bioinformatics.

[31] Hong F., Breitling R., McEntee C.W., Wittner B.S., Nemhauser J.L., Chory J. (2006). RankProd: a bioconductor package for detecting differentially expressed genes in meta-analysis. Bioinformatics.

[32] Schurch N.J., Schofield P., Gierliński M. (2016). How many biological replicates are needed in an RNA-seq experiment and which differential expression tool should you use?. RNA.

[33] Goetz C.G., Tilley B.C., Shaftman S.R. (2008). Movement Disorder Society-sponsored revision of the Unified Parkinson's Disease Rating Scale (MDS-UPDRS): scale presentation and clinimetric testing results. Mov Disord.

[34] Rocca W.A., Maraganore D.M., McDonnell S.K., Schaid D.J. (1998). Validation of a telephone questionnaire for Parkinson's disease. J Clin Epidemiol.

[35] Scherzer C.R., Eklund A.C., Morse L.J. (2007). Molecular markers of early Parkinson’s disease based on gene expression in blood. Proc Natl Acad Sci U S A.

[36] Rai A.K., Muthukumaran N.S., Nisini N. (2024). Transcriptome wide changes in long noncoding RNAs in diabetic ischemic heart disease. Cardiovasc Diabetol.

[37] Fang C., Zhong H., Lin Y. (2018). Assessment of the cPAS-based BGISEQ-500 platform for metagenomic sequencing. Gigascience.

[38] Best M.G., Sol N., In 't Veld S.G.J.G. (2017). Swarm intelligence-enhanced detection of non-small-cell lung cancer using tumor-educated platelets. Cancer Cell.

[39] Li R., Li Y., Kristiansen K., Wang J. (2008). SOAP: short oligonucleotide alignment program. Bioinformatics.

[40] Hong M., Tao S., Zhang L. (2020). RNA sequencing: new technologies and applications in cancer research. J Hematol Oncol.

[41] Langmead B., Salzberg S.L. (2012). Fast gapped-read alignment with bowtie 2. Nat Methods.

[42] Li B., Dewey C.N. (2011). RSEM: accurate transcript quantification from RNA-Seq data with or without a reference genome. BMC Bioinformatics.

[43] Tao Q., Zhang C., Mercier G. (2023). Identification of an APOE ε4-specific blood-based molecular pathway for Alzheimer's disease risk. Alzheimers Dement.

[44] Martirosyan A., Ansari R., Pestana F. (2024). Unravelling cell type-specific responses to Parkinson's Disease at single cell resolution. Mol Neurodegener.

[45] Hu J., Luo Y., Wang X. (2025). Multi-omics analysis of druggable genes to facilitate Alzheimer's disease therapy: a multi-cohort machine learning study. J Prev Alzheimers Dis.

[46] Zhao Y., Mu Y., Zou Y. (2023). Conjoint research of WGCNA, single-cell transcriptome and structural biology reveals the potential targets of IDD development and treatment and JAK3 involvement. Aging.

[47] Johnson E.C.B., Dammer E.B., Duong D.M. (2020). Large-scale proteomic analysis of Alzheimer's disease brain and cerebrospinal fluid reveals early changes in energy metabolism associated with microglia and astrocyte activation. Nat Med.

[48] Zhao W., Langfelder P., Fuller T., Dong J., Li A., Hovarth S. (2010). Weighted gene coexpression network analysis: state of the art. J Biopharm Stat.

[49] Ferrer-Raventós P., Beyer K. (2021). Alternative platelet activation pathways and their role in neurodegenerative diseases. Neurobiol Dis.

[50] Kia D.A., Zhang D., Guelfi S. (2021). Identification of candidate Parkinson disease genes by integrating genome-wide association study, expression, and epigenetic data sets. JAMA Neurol.

[51] Yao S., Zhang X., Zou S.C. (2021). A transcriptome-wide association study identifies susceptibility genes for Parkinson's disease. NPJ Parkinsons Dis.

[52] Li Y.I., Wong G., Humphrey J., Raj T. (2019). Prioritizing Parkinson's disease genes using population-scale transcriptomic data. Nat Commun.

[53] Langmyhr M., Henriksen S.P., Cappelletti C., van de Berg W.D.J., Pihlstrøm L., Toft M. (2021). Allele-specific expression of Parkinson's disease susceptibility genes in human brain. Sci Rep.

[54] Shi J.J., Mao C.Y., Guo Y.Z. (2024). Joint analysis of proteome, transcriptome, and multi-trait analysis to identify novel Parkinson's disease risk genes. Aging.

[55] Zhou S., Tian Y., Song X., Xiong J., Cheng G. (2023). Brain proteome-wide and transcriptome-wide asso-ciation studies, Bayesian colocalization, and Mendelian randomization analyses reveal causal genes of Parkinson's disease. J Gerontol A Biol Sci Med Sci.

[56] Yu E., Larivière R., Thomas R.A. (2024). Machine learning nominates the inositol pathway and novel genes in Parkinson's disease. Brain.

[57] Meléndez-Ramírez C., Cuevas-Diaz Duran R., Barrios-García T. (2021). Dynamic landscape of chromatin accessibility and transcriptomic changes during differentiation of human embryonic stem cells into dopaminergic neurons. Sci Rep.

[58] Wang Z.X., Li Y.L., Pu J.L., Zhang B.R. (2023). DNA damage-mediated neurotoxicity in Parkinson's disease. Int J Mol Sci.

[59] Ghemrawi R., Khair M. (2020). Endoplasmic reticulum stress and unfolded protein response in neurodegenerative diseases. Int J Mol Sci.

[60] Martinez A., Lopez N., Gonzalez C., Hetz C. (2019). Targeting of the unfolded protein response (UPR) as therapy for Parkinson's disease. Biol Cell.

[61] Borsche M., Pereira S.L., Klein C., Grünewald A. (2021). Mitochondria and Parkinson's disease: clinical, molecular, and translational aspects. J Parkinsons Dis.

[62] Li J.L., Lin T.Y., Chen P.L. (2021). Mitochondrial function and Parkinson's disease: from the perspective of the electron transport chain. Front Mol Neurosci.

[63] Nachman E., Verstreken P. (2022). Synaptic proteostasis in Parkinson's disease. Curr Opin Neurobiol.

[64] Shpilka T., Haynes C.M. (2018). The mitochondrial UPR: mechanisms, physiological functions and implications in ageing. Nat Rev Mol Cell Biol.

[65] Spinelli S.L., Casey A.E., Pollock S.J. (2010). Platelets and megakaryocytes contain functional nuclear factor-kappaB. Arterioscler Thromb Vasc Biol.

[66] Yuan H., Liu Y., Zhang J., Dong J.F., Zhao Z. (2023). Transcription factors in megakaryocytes and platelets. Front Immunol.

[67] Beura S.K., Panigrahi A.R., Yadav P., Singh S.K. (2022). Role of platelet in Parkinson's disease: insights into pathophysiology & theranostic solutions. Ageing Res Rev.

[68] Leiter O., Walker T.L. (2019). Platelets: the missing link between the blood and brain?. Prog Neurobiol.

[69] Schroer A.B., Ventura P.B., Sucharov J. (2023). Platelet factors attenuate inflammation and rescue cognition in ageing. Nature.

[70] Giles J.A., Greenhalgh A.D., Denes A. (2018). Neutrophil infiltration to the brain is platelet-dependent, and is reversed by blockade of platelet GPIbα. Immunology.

[71] Chen H., Teng Y., Chen X. (2021). Platelet-derived growth factor (PDGF)-BB protects dopaminergic neurons *via* activation of Akt/ERK/CREB pathways to upregulate tyrosine hydroxylase. CNS Neurosci Ther.

[72] Chao J., Yang L., Yao H., Buch S. (2014). Platelet-derived growth factor-BB restores HIV tat-mediated impairment of neurogenesis: role of GSK-3β/β-catenin. J Neuroimmune Pharmacol.

[73] Martínez-Iglesias O., Naidoo V., Cacabelos N., Cacabelos R. (2021). Epigenetic biomarkers as diagnostic tools for neurodegenerative disorders. Int J Mol Sci.

[74] Rachinger N., Fischer S., Böhme I. (2021). Loss of gene information: discrepancies between RNA sequencing, cDNA microarray, and qRT-PCR. Int J Mol Sci.

[75] Everaert C., Luypaert M., Maag J.L.V. (2017). Benchmarking of RNA-sequencing analysis workflows using whole-transcriptome RT-qPCR expression data. Sci Rep.

[76] Knorr D.Y., Rodriguez Polo I., Pies H.S. (2023). The cytokine receptor CRLF3 is a human neuroprotective EV-3 (Epo) receptor. Front Mol Neurosci.

[77] Wegscheid M.L., Anastasaki C., Hartigan K.A. (2021). Patient-derived iPSC-cerebral organoid modeling of the 17q11.2 microdeletion syndrome establishes CRLF3 as a critical regulator of neurogenesis. Cell Rep.

[78] Bennett C., Lawrence M., Guerrero J.A. (2022). CRLF3 plays a key role in the final stage of platelet genesis and is a potential therapeutic target for thrombocythemia. Blood.

[79] Bowles K.R., Pugh D.A., Liu Y. (2022). 17q21.31 sub-haplotypes underlying H1-associated risk for Parkinson's disease are associated with LRRC37A/2 expression in astrocytes. Mol Neurodegener.

