# Supplementary material for: Identification of platelet-derived biomarker in a cohort of Parkinson's disease
Source: Genes Dis. 2026 Apr 2;14(2):102171. doi: 10.1016/j.gendis.2026.102171 (PMC13677027; doi:10.1016/j.gendis.2026.102171)
Supplement: Supplementary file 1 [file mmc1.docx]

**Figure S1** Flow chart of sample selection and grouping. Quality of PLT RNA samples were determined before RNA-sequencing. Revised diagnosis and grouping of PD and APD were made prior to final data analysis. Samples mainly included for analysis and for later verification are shown on the right and left, respectively.

**Figure S2** Comparative RNA analysis reveals high purity of PLT samples. **(A)** Average number of the detected genes in the PLTs of patients and controls, with no overall difference among the groups. **(B)** The correlation matrix of genes in PLT samples and publicly available data that include two datasets of PLT (GSE68086, GSE89843) and one dataset of 7 types of immune cells (GSE62408). Pearson correlation analysis was used to analyze the similarity of expression profiles among different cells. This result revealed that PLTs samples were without significant contaminations.

**Figure S3** Top module genes with high connectivity and DEG values in the SN. Top, in each module that is significantly correlated with PD traits, top 20 genes that are interconnected with maximum numbers using Maximal Clique Centrality (MCC) are plotted by Cytoscape. Bottom, alterations in gene expression level (|Log2FC|) of the top 20 genes in SN of PD relative to HC. Dark blue, significantly downregulated; Dark Red significantly upregulated. Light blue/red, no significance.

**Figure S4** Top module genes with high connectivity and DEG values in PLT. MCC was used to plot the top 20 genes that are highly interconnected in each module. Bottom, alterations in gene expression level (|Log2FC|) of the top 20 genes in PLT of PD relative to HC. Dark blue, significantly downregulated; Light blue, no significance.

**Figure S5** The mechanisms of neuronal cells in SN neurons transcriptional data and gene expression trends. **(A)** The transcriptional data of neuronal cells in SN neurons were extracted and subjected to WGCNA analysis. The PD phenotype has a significant correlation with the Mediumpurple3, Sienna3 and Darkgrey modules (P≤0.05). (**B–D)** The main enriched GO-BP pathways of the genes contained in those 3 modules. GO-BP pathways included the well-documented dopamine biosynthesis (B) and synaptic signaling (D). Notably, pathways such as regulation of chromosome separation (B) and mitotic sister chromatid segregation (C) were also identified, suggesting that the altered chromosomal processing were preserved in both PLTs and the SN neurons.

**Figure S6** Combination of CRLF3, TBXA2R and LRRC37A2 genes shows the best diagnostic performance. **(A)** ROC of 20 individual genes to identify HC and PD. AUC > 0.7 was considered to have good diagnostic efficacy. **(B)** ROC of PD vs. HC using a combination of CRLF3, TBXA2R and LRRC37A2. c ROC of PD vs. APD using a combination of CRLF3, TBXA2R and LRRC37A2. d ROC of HC vs. APD using a combination of CRLF3, TBXA2R and LRRC37A2.
